# Supplementary material for: Doping of Transparent Electrode Based on Oriented Networks of Nickel in Poly(3,4-Ethylenedioxythiophene) Polystyrene Sulfonate Matrix with P-Toluenesulfonic Acid
Source: Nanomaterials (Basel). 2023 Feb 23;13(5):831. doi: 10.3390/nano13050831 (PMC10005722; doi:10.3390/nano13050831)
Supplement: Supplementary file 1 [file nanomaterials-13-00831-s001.zip › nanomaterials-2153256-supplementary.pdf]

nanomaterials

Supplementary Materials

Doping of Transparent Electrode Based on Oriented Networks of Nickel in Poly(3,4-Ethylenedioxythiophene) Polystyrene Sulfonate Matrix with P-Toluenesulfonic Acid

Irek R. Nizameev [1,2,*], Guliya R. Nizameeva [1,3] and Marsil K. Kadirov [1,3]

1 Arbuzov Institute of Organic and Physical Chemistry, FRC Kazan Scientific Center, Russian Academy of Sciences, Arbuzov Str. 8, Kazan 420088, Russia
2 Department of Nanotechnology in Electronics, Kazan National Research Technical University named after A.N. Tupolev—KAI, 10, K. Marx str., Kazan 420111, Russia
3 Department of Physics, Kazan National Research Technological University, 68, K. Marx str., Kazan 420015, Russia
* Correspondence: inizameyev@iopc.ru

S1. Investigation of the Chemical Reduction Reaction Stages to Determine the Time During Which All the Nickel in the Solution Is Reduced

Studies of the obtained metal networks and by-products were carried out by X-ray fluorescence analysis (XRF) separately. The nickel lines absence in the spectrum of the reaction by-products meant that all the nickel from the salt passed into the metallic state. Figure S3 shows the spectrum of reaction by-products obtained after 1 h of synthesis. The spectrum clearly shows the nickel lines $L\alpha1$ = 0.85 keV, $K\alpha1$ = 7.48 keV, the chlorine line $K\alpha1$ = 2.62 keV, and the sodium line $K\alpha1$ = 1.04 keV. In addition to these lines, the spectrum contains lines corresponding to the substrate on which the reaction products were studied: the carbon line ($K\alpha1$ = 0.28 keV) and copper lines ($L\alpha1$ = 0.93 keV, $K\alpha1$ = 8.05 keV, $K\beta1$ = 8.90 keV). Based on these data, it can be concluded that the $NiCl_2{\cdot}6H_2O$ salt did not react completely. A series of experiments with different durations of the nickel reduction chemical reaction made it possible to determine the optimal reaction time is 5 h at a temperature of 70 °C (Figure S5). Since nickel can be fixed to surfaces using a magnetic field, it is easy to get rid of the by-products of the reaction. This phenomenon is what we used in our work. However, it is essential to wash the synthesized product thoroughly. We concluded that using deionized water and isopropyl alcohol (or ethyl alcohol) is sufficient. The XRF method determined the degree of obtained material purity by the absence of the reaction by-products lines (Figure S5). In addition to the absence of undesirable chemical elements in the synthesized material, it is important to make sure that nickel is represented precisely by fibres. The presence of nanoparticles and their other forms is minimized. For this, the method of transmission electron microscopy (TEM) was used. After each synthesis, a control sample was prepared in the form of a suspension of nickel in isopropanol. For statistical validity, five samples for TEM were taken from the control sample. The formation of nickel in the form of separate particles after carrying out the complete reaction (5 h of synthesis) is negligible (significantly less than 1% by weight). The shape and dimensions of submicron fibres have been studied previously. Here we can add the fact that during long-term synthesis (reaction time is 5 h), the shape of the fibres is transformed to a more developed one with the formation of a significant number of "thorns" on the surface (Figure S6). This fact may be of interest for applications where the specific surface area of the material plays an important role (for example, heterogeneous catalysis).

S2. Figures

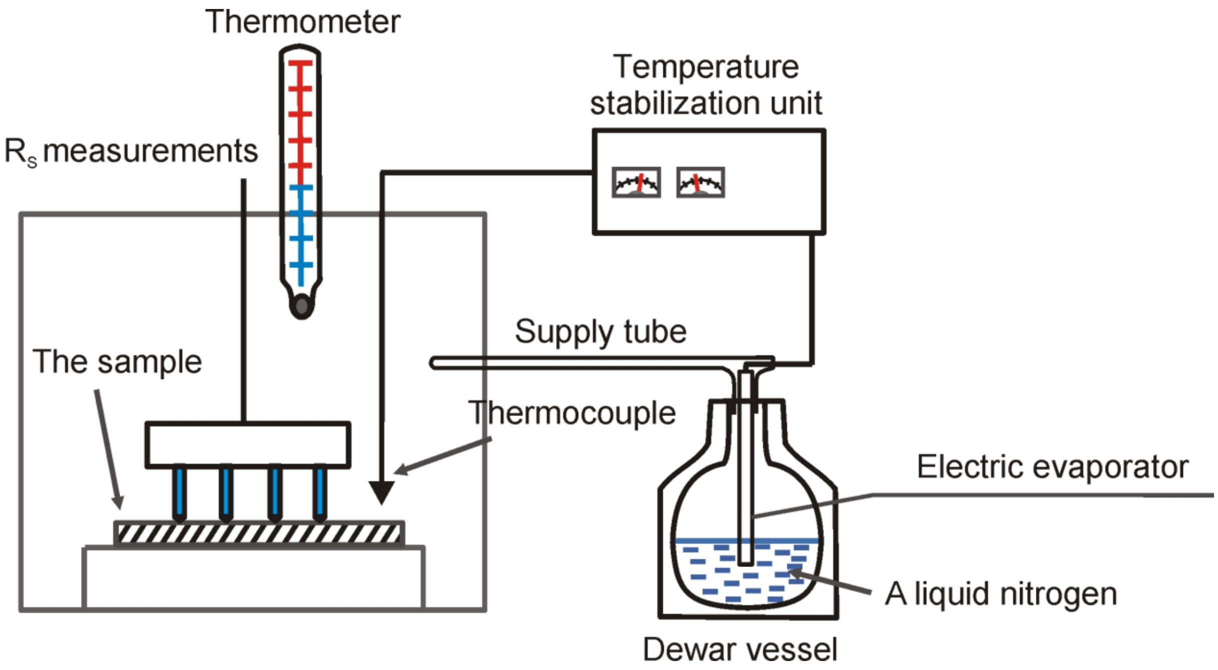

Figure S1. Schematic illustration of the temperature regulation and stabilization system.

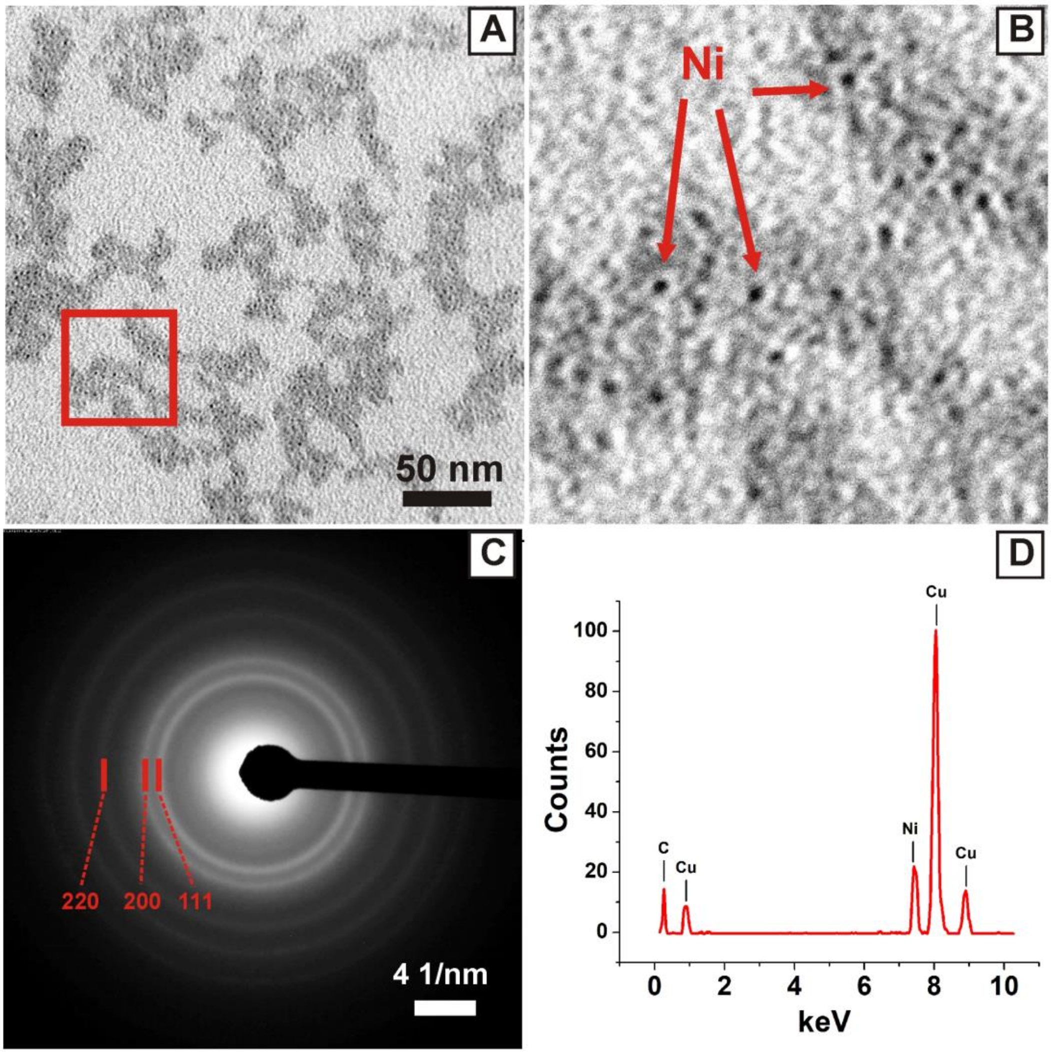

Figure S2. TEM images of nickel nanoparticles formed as a result of incomplete synthesis (**A**,**B**); the microdiffraction pattern corresponds to the face-centered cubic lattice of nickel (**C**); spot microanalysis XRF spectrum corresponding to the formed nanoparticles (lines of carbon and copper refer to the substrate on which the samples were studied) (**D**).

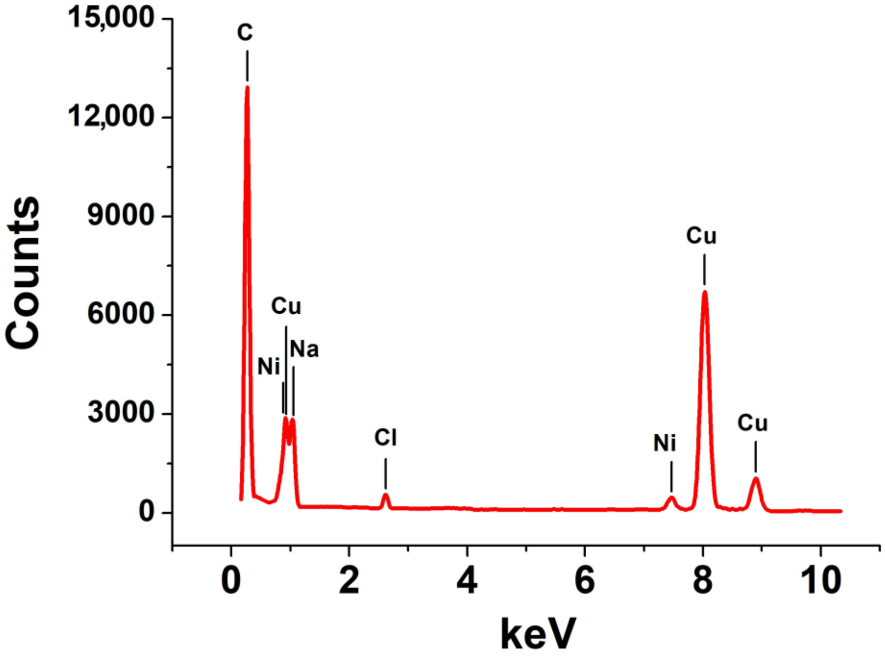

Figure S3. TEM-EDX spectrum of the reaction by-products obtained after 1 h of synthesis.

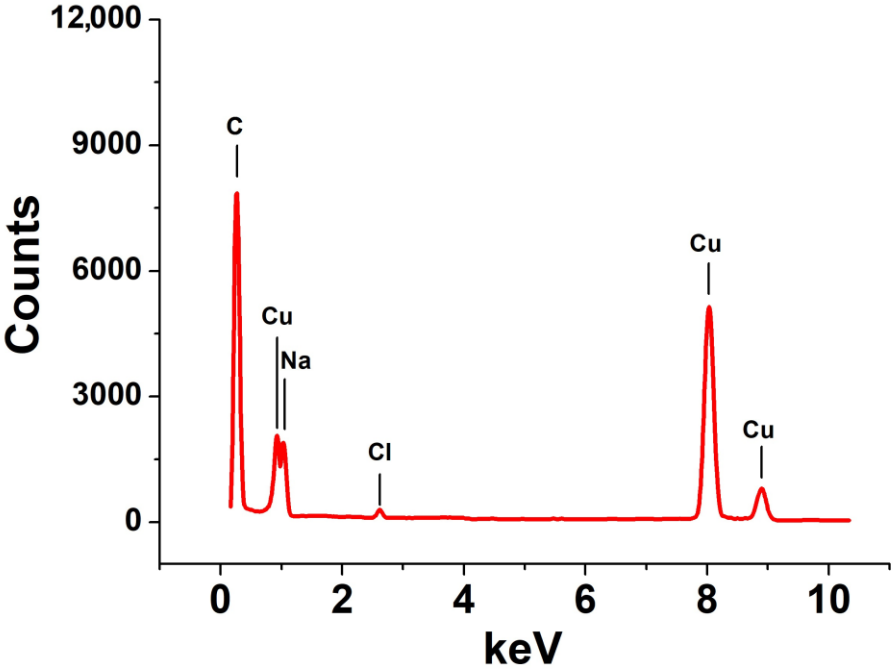

Figure S4. TEM-EDX spectrum of the reaction by-products obtained after 5 h of synthesis.

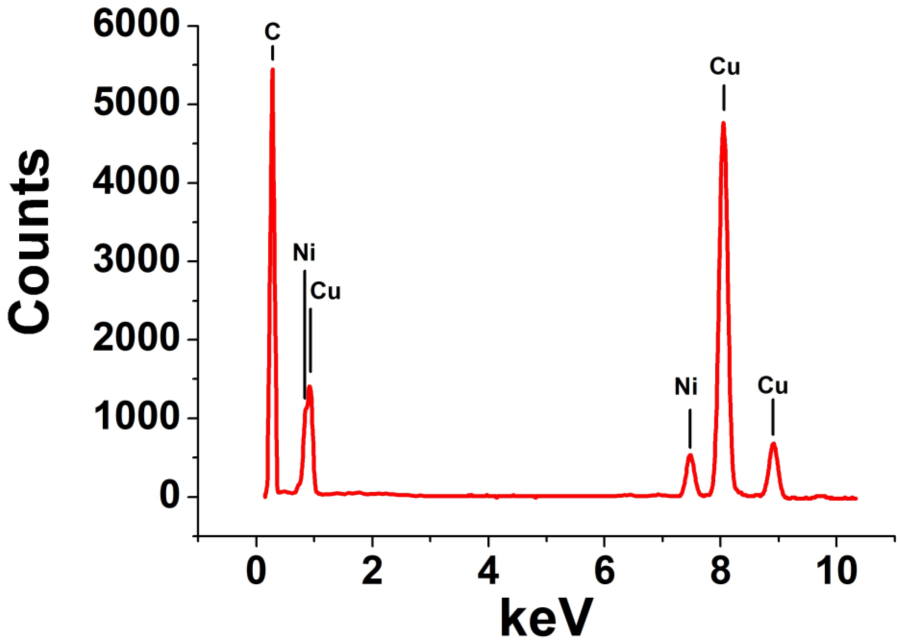

Figure S5. TEM-EDX spectrum of the material obtained in the reaction (metal nickel).

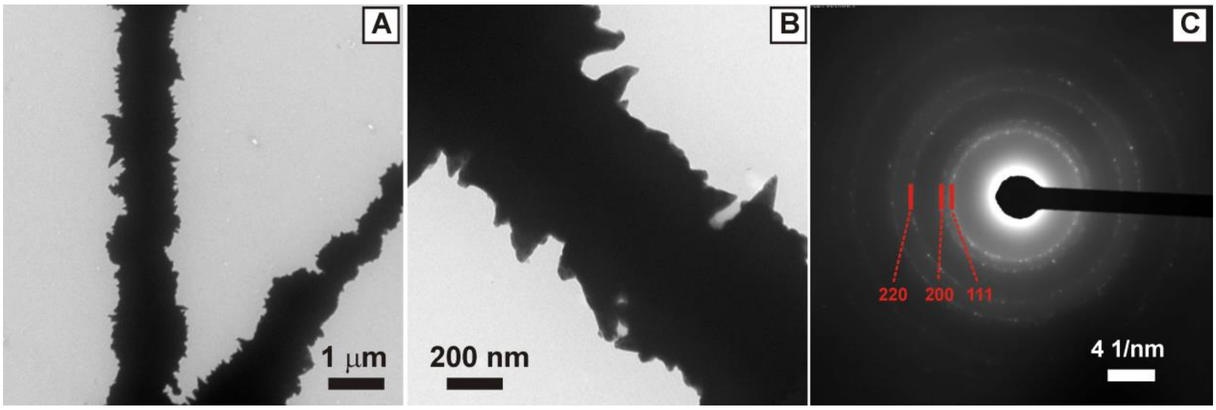

Figure S6. TEM images of nickel fibres formed during full synthesis (**A**,**B**); microdiffraction pattern corresponding to synthesized fibres (**C**).

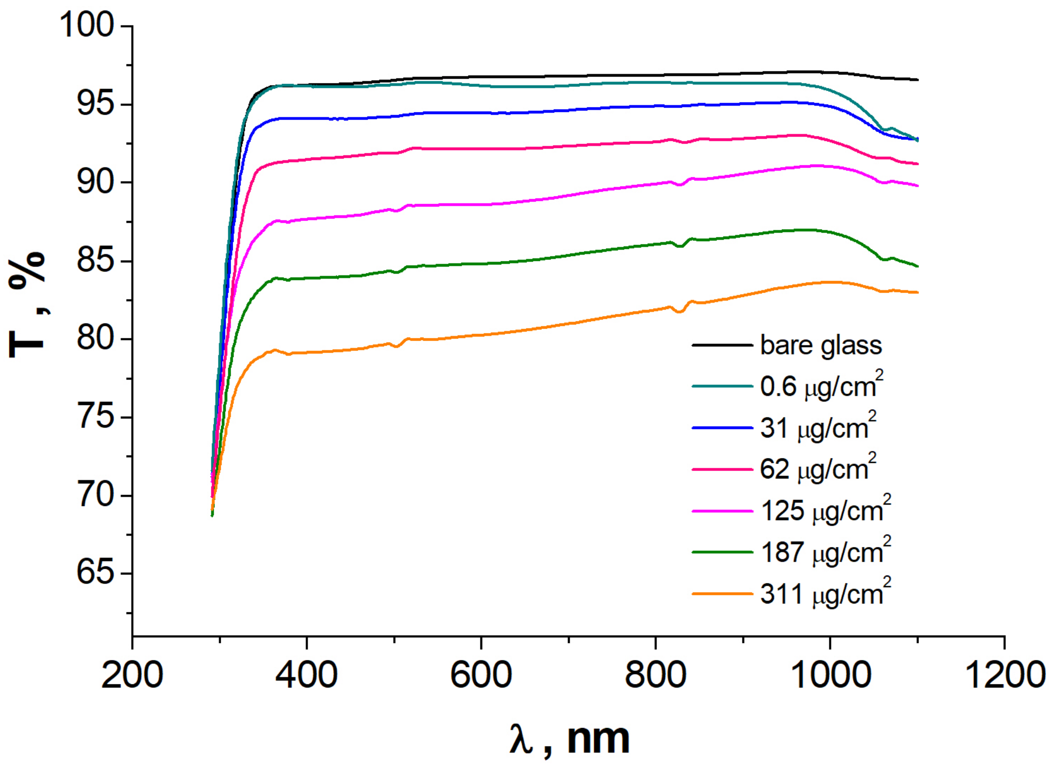

Figure S7. Transmission spectra of oriented nickel networks on a glass substrate in the UV, visible, and near-IR regions for different amounts of deposited metal.

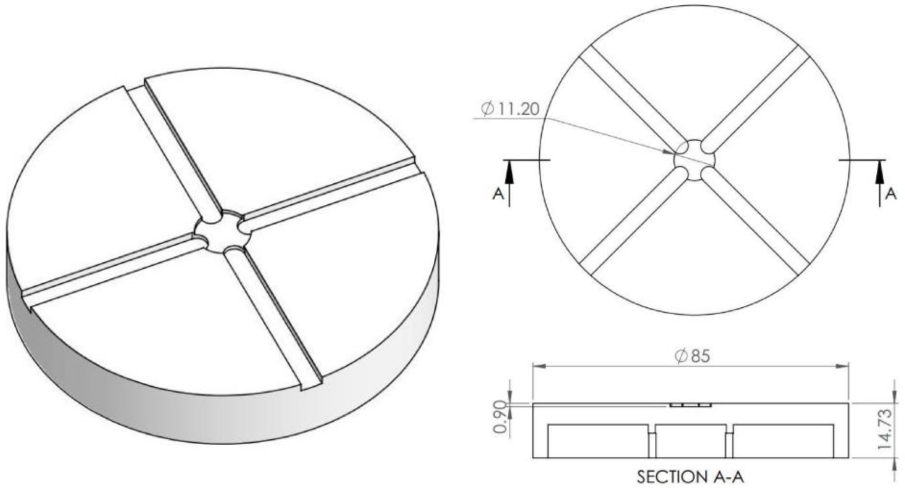

Figure S8. Drawing of a removable cartridge-holder of the Ossila Spin Coater.

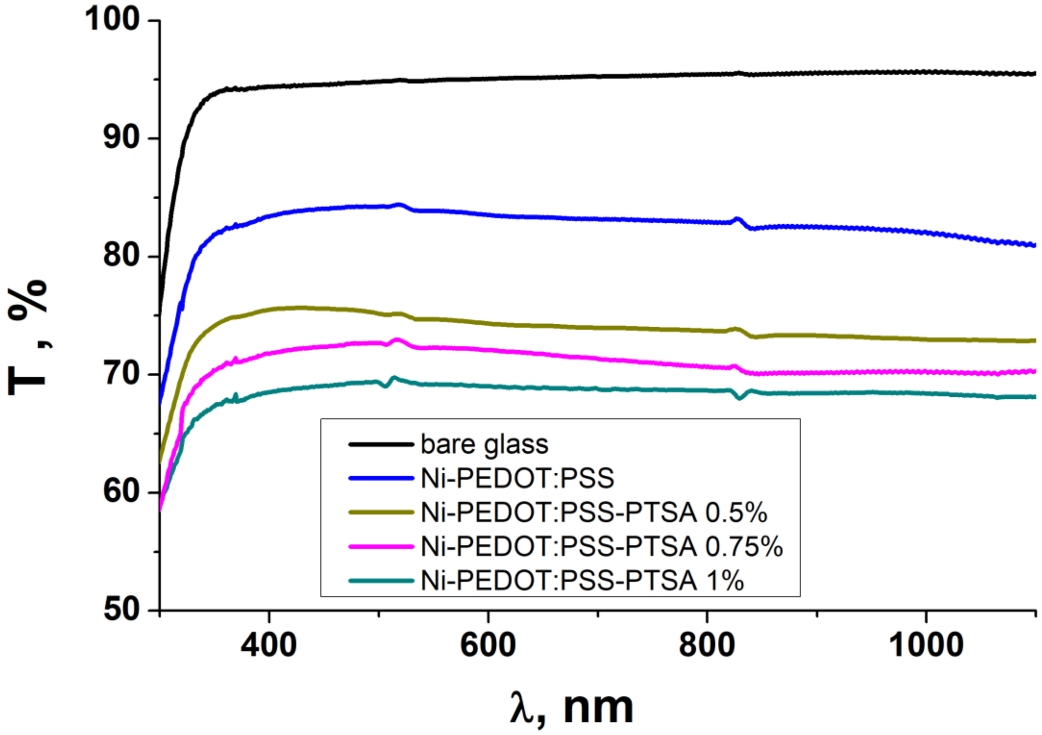

Figure S9. Transmission spectra of modified {Ni + PEDOT: PSS + PTSA} coatings on a glass substrate in the UV, visible, and near-IR regions corresponding to different concentrations of PTSA in an aqueous PEDOT: PSS dispersion.
